# Screening of Korean Medicinal Plant Extracts for *α*-Glucosidase Inhibitory Activities

**Published:** 2011

**Authors:** Shruti Sancheti, Sandesh Sancheti, Seung-Hun Lee, Jae-Eun Lee, Sung-Yum Seo

**Affiliations:** a *Department of Biology, Kongju National University, Kongju 314-701, Korea.*; b *Korean Collection of Herbal Extract, Inc., Kongju 314-701, Korea.*

**Keywords:** *α*-Glucosidase inhibitor, Korean plants, Screening, Glycosidases

## Abstract

Glycosidases are the enzymes involved in various biochemical processes related to metabolic disorders and diseases. Therefore, much effort has been focused on searching novel pharmacotherapy for the treatment of these ailments from medicinal plants due to higher safety margins. To pursue these efforts, the present study was performed to evaluate the *α*-glucosidase inhibitory activities of thirty Korean medicinal plant extracts. Among the plants studied, *Euonymus sachalinensis*,* Rhododendron schlippenbachii*,* Astilbe chinensis* and* Juglans regia *showed the strongest *α*-glucosidase inhibitory activity with IC_50_ values of 10, 20, 30 and 80 µg/mL, respectively. In addition, *Meliosma oldhamii *and *Symplocos chinensis *showed moderate *α*-glucosidase inhibition with IC_50_ values of 150 and 220 µg/mL, respectively. Therefore, they might prove to be a potential natural source for the treatment of metabolic ailments such as, diabetes, and need further investigations.

## Introduction

Glycosidases are widespread in microorganisms, plants, and animals. They are a very important class of enzymes, which catalyze a hydrolytic cleavage of glycosidic bonds in oligosaccharides or glycoconjugates. Among these glycosidases, *α*-glucosidase is able to catalyze the cleavage of glycosidic bonds involving terminal glucose connected at the site of cleavage through *α*-linkage at the anomeric center (1-3).

Glycosidases are involved in several important biological processes (like: digestion, biosynthesis of glycoproteins and lysosomal catabolism of glucoconjugates) related to metabolic disorders and diseases, such as, diabetes,obesity,glycosphingolipid lysosomal storage disease,HIV infections,and tumors (1-3). These observations indicate that the inhibition of glycosidases would represent a novel pharmacological approach towards the treatment of the above mentioned complications, including diabetes. 

Diabetes mellitus (DM) is a metabolic disorder characterized by chronic hyperglycemia that has a significant impact on the health, quality of life and life expectancy of patients, as well as health care system (1, 4). In clinical practice, the DM treatment is restricted to the use of oral hypoglycemic agents and insulin, where the former possesses serious side effects (5). Therefore, many traditional herbal remedies for treating diabetes used throughout the world as plant drugs and herbal formulations, are frequently considered to be free from side effects and less toxic than the synthetic one (6). 

**Table 1:** *α*-Glucosidase inhibitory activities and IC_50_ values of the studied Korean plant extracts

**Sr. No.**	**Plant name**	**Family**	**Plant part**	***α*** **-glucosidase inhibition**	
				**% Inhibition**	**IC**_50_ ** Values (µg/mL)** ^a^
1	Actinidia arguta	Actinidiaceae	Whole plant	33 ± 1	--
2	Meliosma oldhamii	Sabiaceae	Whole plant	67 ± 3	150 ± 3
3	Aster tataricus	Asteraceae	Stem	n. a.	--
4	Capsella bursa-pastoris	Brassicaceae	Whole plant	23 ± 1	--
5	Allium macrostemon	Alliaceae	Whole plant	19 ± 2	--
6	Rhododendron schlippenbachii	Ericaceae	Leaf	98 ±1	20 ± 1
7	Pyrola japonica	Pyrolaceae	Whole plant	29 ± 2	--
8	Symplocos chinensis	Symplocaceae	Leaf	52 ± 2	220 ± 5
9	Juglans regia	Juglandaceae	Whole plant	99 ± 1	80 ± 2
10	Sinapsis alba	Brassicaceae	Seed	22 ± 2	--
11	Aster tataricus	Asteraceae	Root	17 ± 3	--
12	Magnolia kobus	Magnoliaceae	Whole plant	4 ± 1	--
13	Inula helenium	Asteraceae	Whole plant	9 ± 2	--
14	Digitaria violascens	Poaceae	Whole plant	n. a.	--
15	Dendropanax morbifera	Araliaceae	Whole plant	39 ± 3	--
16	Sesamum indicum	Pedaliaceae	Whole plant	11 ± 2	--
17	Alisma canaliculatum	Alismataceae	Rhizomes	22 ± 3	--
18	Celtis sinensis	Cannabaceae	Whole plant	25 ± 1	--
19	Astilbe chinensis	Saxifragaceae	Rhizomes	95 ± 2	30 ± 2
20	Corydalis remota	Papaveraceae	Whole plant	23 ± 2	--
21	Phlomis umbrosa	Labiatae	Whole plant	12 ± 2	--
22	Curcuma zedoaria	Zingiberaceae	Whole plant	27 ± 2	--
23	Gleditsia japonica	Fabaceae	Whole plant	n. a.	--
24	Miscanthus sinensis	Poaceae	Whole plant	30 ± 3	--
25	Heracleum moellendorffii	Apiaceae	Whole plant	17 ± 2	--
26	Draba nemorosa	Brassicaceae	Whole plant	11 ± 1	--
27	Vaccinium hirtum	Ericaceae	Whole plant	46 ± 1	--
28	Smilax sieboldii	Smilacaceae	Whole plant	24 ± 3	--
29	Euonymus sachalinensis	Celastraceae	Leaf	99 ± 1	10 ± 1
30	Sinomenium acutum	Menispermaceae	Bark	28 ± 2	--

Note: The IC_50_ values of 1,2,3,4,6-penta-O-galloyl-*β*-D-glucose (positive control) for *α*-glucosidase inhibitory activity was measured as 0.37 ± 0.03 µg/mL, respectively.

^a^ All results are represented as mean ± SD (n = 3).

n. a.: no activity. --: Not determined (IC_50 _values were only determined for the plant extracts with ≥ 50% inhibition at 5 mg/mL).

Thus, many plants and crude drugs have recently been tested for their effects on α-glucosidase inhibition.

To pursue the findings, in this research, we screened thirty Korean medicinal plants. The details of the plants’ scientific names and families are listed in Table 1. A literature survey did not show any reference to a previous work on theα-glucosidase inhibitory activities of these thirty plant extracts.

## Experimental

Plant material

The dried and matured plant parts of thirty Korean medicinal herbs were obtained from “Korean Collection of Herbal Extracts” a Biotech company in Korea. A collection of voucher specimen is available with the company (Korea Collection of Herbal Extracts, 2000).

Extraction

The dried plant parts individually were chopped into small pieces and pulverized into a fine powder. The powdered plant materials (100 g, dry weight) were kept for extensive decoction in 80% methanol for 3 days at room temperature. The extracts were then concentrated using rotary vacuum evaporator at 20-30°C to obtain the dried crude extracts.

Reagents

α-Glucosidase (from Saccharomyces cerevisiae type I) and 4-nitrophenyl α-D-glucopyranoside were purchased from Sigma-Aldrich (St. Louis, MO, USA). Other commercially available reagents and solvents were used as received.

α-Glucosidase assay

The enzyme inhibition activity for α-glucosidase was evaluated according to the method previously reported by Shibano et al. (7) with minor modifications. The reaction mixture consisted of 50 μL of 0.1 M phosphate buffer (with pH of 7.0), 25 μL of 0.5 mM 4-nitrophenyl α-D-glucopyranoside (dissolved in 0.1 M phosphate buffer, with pH of 7.0), 10 μL of test sample and 25 μL of α-glucosidase solution (a stock solution of 1 mg/mL in 0.01 M phosphate buffer, with pH of 7.0 was diluted to 0.1 Unit/mL with the same buffer, with pH of 7.0 just before assay). This reaction mixture was then incubated at 37°C for 30 min. Then, the reaction was terminated by the addition of 100 μL of 0.2 M sodium carbonate solution. The enzymatic hydrolysis of substrate was monitored by the amount of p-nitrophenol released in the reaction mixture at 410 nm using microplate reader. Individual blanks were prepared for correcting the background absorbance, where the enzymes were replaced with buffer. Controls were conducted in an identical manner replacing the plant extracts with methanol. 1, 2, 3, 4, 6-penta-O-galloyl-β-D-glucose was used as positive control. All experiments were carried out in triplicates. The inhibition percentage of α-glucosidase was assessed by the following formula:

I α-glucosidase% = 100 X (ΔA_Control _- ΔA_Sample_) / _ΔAControl_

ΔA_Control_ = ΔA_Test_ - ΔA_Blank_

ΔA_Sample_ = ΔA_Test_ - ΔA_Blank_

Statistical analyses

All assays were performed at least three times with triplicate samples. All results are expressed as mean ± SD. IC_50 _values were only determined for the plant extracts with inhibition ≥ 50% at 5 mg/mL by plotting a percent inhibition versus concentration curve, in which the concentration of sample required for 50% inhibition was determined and expressed as IC_50 _value.

## Results and Discussion

Plants have always been used as an exemplary source of drugs and many of the currently available drugs have been directly or indirectly derived from them (8). Many herbal extracts are being used in the preparation of advanced remedies for diabetes, in which α-glucosidase inhibitors play an important role by controlling postprandial blood glucose levels by means of retarding uptake of dietary carbohydrates (9). Therefore, in search of such potent α-glucosidase inhibitors from natural source, in the present study thirty Korean medicinal plant extracts have been evaluated for their α-glucosidase inhibitory activity and compared with 1, 2, 3, 4, 6-penta-O-galloyl-β-D-glucose as a positive control. 

In this study, the α-glucosidase inhibitory activity of the thirty plant extracts was evaluated at 5 mg/mL concentration at the preliminary level and the percentage inhibitions are shown in Table 1. 

The present data revealed that, six plant extracts demonstrated α-glucosidase inhibition ≥ 50%, namely, Euonymus sachalinensis, Rhododendron schlippenbachii, Astilbe chinensis, Juglans regia, Meliosma oldhamii and Symplocos chinensis with IC_50_ values of 10 ± 1, 20 ± 1, 30 ± 2, 80 ± 2, 150 ± 3 and 220 ± 5 µg/mL, respectively. The traditional uses of these plants are listed in Table 2. These active plants have no documentary evidence in the literature for their *α*-glucosidase inhibitory potency.

**Table 2:** Details of the traditional indications of the active plant extracts

**Sr. no.**	**Plant name**	**Traditional indications**
1	***Meliosma oldhamii ***	Treatment of ailments of the liver
2	***Rhododendron schlippenbachii***	Discharge of cardiotonic phlegm
3	***Symplocos chinensis***	Cold, fever, malaria, relief of cough, detoxification
4	Juglans regia	Anti-inflammatory, astringent, anticancer, blood purifier, laxative, diuretic, anthelmintic
5	Astilbe chinensis	Anti-inflammatory, anticancer, hepatoprotective, treating arthralgia, chronic bronchitis, headache
6	***Euonymus sachalinensis ***	Treatment of stomachalgia

In addition, in the present screening, eight plant extracts showed medium activity, ranging from 25 to 49%, 13 plant extracts showed less than 25% inhibition, and 3 plant extracts did not exhibit *α*-glucosidase inhibitory activity (Table 1).

The *α*-glucosidase inhibitory potential of the identified potent crude extracts was lower as compared to PGG (Table 1), since crude extracts contain non-active components along withthe active ones. Therefore, the crude extracts of *Euonymus sachalinensis*,* Rhododendron schlippenbachii*,* Astilbe chinensis*, *Juglans regia*, *Meliosma oldhamii *and *Symplocos chinensis* seem to be relatively potent inhibitors, where the inhibitory activity could further be improved by separation and purification of the active components.

Thus, introduction of such innovative herbal extracts for the treatment of diabetes and other related metabolic disorders, where the *α*-glycosidase inhibition plays a key role, may prove fortune. However, further *in-vivo*studies needed to be confirmed to provide strong biochemical rationale.
